# Calprotectin as a Diagnostic Marker for Lower Respiratory Tract Infection and Sepsis in the Emergency Department

**DOI:** 10.1093/ofid/ofag331

**Published:** 2026-06-03

**Authors:** Martin Wollmer, Torgny Wessman, Olle Melander, Anders Larsson, Toralph Ruge

**Affiliations:** Department of Emergency and Internal Medicine, Skåne University Hospital, Malmö, Sweden; Department of Clinical Sciences Malmö, Lund University, Lund, Sweden; Department of Emergency and Internal Medicine, Skåne University Hospital, Malmö, Sweden; Department of Clinical Sciences Malmö, Lund University, Lund, Sweden; Department of Clinical Sciences Lund, Lund University, Lund, Sweden; Department of Emergency and Internal Medicine, Skåne University Hospital, Malmö, Sweden; Department of Clinical Sciences Malmö, Lund University, Lund, Sweden; Department of Medical Sciences, Uppsala University, Uppsala, Sweden; Department of Emergency and Internal Medicine, Skåne University Hospital, Malmö, Sweden; Department of Clinical Sciences Malmö, Lund University, Lund, Sweden

**Keywords:** diagnosis, emergency service, leukocyte L1 antigen complex, respiratory tract infections, sepsis

## Abstract

**Background:**

Calprotectin, a neutrophil activation marker, is a promising diagnostic biomarker for infection and sepsis, but its usefulness in the emergency department (ED) is unclear. The aim of this study was to investigate the diagnostic value of calprotectin for sepsis and the source of infection in the ED.

**Methods:**

A total of 583 prospectively included patients presenting with suspected sepsis to the ED of a university hospital in southern Sweden were analyzed. Calprotectin was measured in plasma samples obtained at admission.

**Results:**

Mean age was 69 years and 49% were female. Calprotectin discriminated between sepsis and noninfectious systemic inflammatory response syndrome with an area under the curve (AUC) of 0.63, but was not an independent predictor in multivariable analysis including C-reactive protein (CRP). The Modified Early Warning Score did not show any discriminatory ability. Calprotectin and CRP discriminated between lower respiratory tract infection and (1) upper respiratory tract infection, (2) urinary tract infection, and (3) other sources of infection. Addition of calprotectin to CRP significantly increased the AUC for lower versus upper respiratory tract infection and lower respiratory tract infection versus urinary tract infection. Calprotectin was an independent predictor for all outcomes while CRP was only an independent predictor of lower versus upper respiratory tract infection.

**Conclusions:**

Calprotectin may improve differentiation of lower respiratory tract infections from upper respiratory tract infections and urinary tract infections in patients presenting to the ED with sepsis. Further research is needed to clarify if calprotectin adds diagnostic value to standard clinical assessment.

## Teaser

Calprotectin at emergency department admission improves the diagnostic performance of C-reactive protein alone to identify the lower respiratory tract as the source of infection in patients with sepsis. This suggest that calprotectin may aid clinicians in choosing empirical antibiotic therapy.

Sepsis is a life-threatening condition affecting over 30 million people and causing more than 5 million deaths every year [1]. Early antibiotic therapy is fundamental to improve outcomes [2], but empiric treatment must be balanced with the risk of increasing antibiotic resistance [3]. Because sepsis is a syndrome without a gold standard diagnostic test [4], early recognition remains difficult. Clinical criteria have so far been insufficient for sepsis detection in the emergency department (ED). The Systemic Inflammatory Response Syndrome (SIRS) is nonspecific [5], while the more recent Quick Sequential Organ Failure Assessment (qSOFA) score has inferior sensitivity to SIRS [6]. The Modified Early Warning Score (MEWS) has higher sensitivity than qSOFA but is also insufficient as a single tool for sepsis detection [7].

Biomarkers may improve early sepsis detection, yet none are currently recommended to guide initiation of antibiotic therapy [8]. C-reactive protein (CRP) is widely used in clinical practice but increases relatively late after inflammatory stimuli, typically peaking around 48 hours [9]. Calprotectin, a neutrophil activation marker abundantly present in the cytoplasm of neutrophils [10], peaks around 5 hours after endotoxemia challenge [11]. It has shown promise as a diagnostic marker of sepsis in the intensive care unit (ICU) [12] and for differentiating bacterial from viral infections [13]. However, the diagnostic value of calprotectin in the ED remains unclear.

The aim of this study was to investigate the diagnostic value of calprotectin for sepsis and the source of infection in patients presenting to the ED with suspected sepsis.

## METHODS

### Study Population

This was a secondary analysis of the ED-Sepsis cohort. ED-Sepsis was a prospective observational cohort study conducted at the ED of Skåne University Hospital, Malmö, Sweden, a tertiary academic center [14]. Inclusion criteria were suspected infection, as judged by the research nurse, and fulfilment of at least 2 of the following SIRS criteria: (1) body temperature >38°C or <36°C, or self-reported fever/chills <24 hours; (2) respiratory rate >20 breaths/minute; and (3) heart rate >90 beats/minute. White blood cell count was not used as an inclusion criterion as it was not available during screening. Age <18 years was the only exclusion criterion. Inclusion was made by attending research nurses during office hours (6 Am to 6 Pm) between 1 December 2013 and 1 February 2015.

### Clinical Parameters

Information on demographics and comorbidities was retrieved from the electronic medical records. An infectious disease physician systematically reviewed the electronical medical records to establish infectious diagnosis. In ambiguous cases, another 2 infectious disease physicians independently reviewed the records and reached a classification. Results of CRP analysis, but not calprotectin, were available to the reviewing physicians. For analysis, the source of infection was categorized as lower respiratory tract infection (LRTI), upper respiratory tract infection (URTI), urinary tract infection (UTI), or other source of infection (distribution is presented in Supplementary Table 1). Blood pressure, oxygen saturation, and heart rate were measured with a fully automated oscillometric device (CARESCAPE Monitor B850 or B650, General Electric Healthcare, Danderyd, Sverige). Respiratory rate was calculated manually by an ED nurse. Level of consciousness was categorized as alert, verbal, pain, or unresponsive. MEWS was calculated and dichotomized at ≥5 according to Subbe et al [15]. Ethylenediaminetetraacetic acid (EDTA) plasma samples were drawn within 1 hour of admission to the ED, frozen within 2 hours of collection, and stored at −80°C until analysis.

### Outcomes

The study had 2 outcomes: (1) discrimination between sepsis and noninfectious SIRS in patients with suspected sepsis, and (2) discrimination between LRTI and (*i*) URTI, (*ii*) UTI, or (*iii*) other sources of infection, in patients with sepsis.

### Biochemical Analyses

Calprotectin was analyzed in EDTA plasma samples with particle-enhanced turbidimetric assay (PETIA) on a Mindray BS380 chemistry analyzer (Mindray, Shenzhen, China) using calprotectin reagents from Gentian AS (Moss, Norway).

CRP was analyzed according to local routine with PETIA on Roche Cobas c501, c502, or c701 analyzers (Roche Diagnostics, Solna, Sweden).

### Ethical Considerations

The study was performed in accordance with the Declaration of Helsinki and approved by the Regional Ethical Review Board in Lund, Sweden (reference number 2013/635). Written and oral informed consent was obtained from all patients or their next of kin.

### Statistical Analysis

Normally distributed variables were presented as mean value with standard deviation (SD), skewed variables as median value with interquartile range (IQR), and categorical variables as number with proportion (%). The *t*-test, Mann–Whitney *U* test, or χ^2^ test were used to test for differences between 2 groups. Kruskal–Wallis 1-way analysis of variance with Bonferroni correction for multiple tests was used to test for differences between >2 groups. Missing values for individual parameters of MEWS were considered to be normal (ie, 0 points). MEWS was analyzed as a binary variable dichotomized at ≥5. Correlations between continuous variables were tested by Spearman rank correlation. In the analyses of source of infection, the outcome LRTI was tested against URTI, UTI, and other sources of infection, respectively. Receiver operating characteristic (ROC) analysis and Youden index were used to identify optimal cut-off points for calprotectin and CRP. The diagnostic abilities at the optimal cut-off points were estimated by ROC analysis. To assess the added value of calprotectin to CRP, the predicted probabilities of CRP and the combination of CRP and calprotectin were obtained by binary logistic regression analysis and then tested by ROC analysis. Multivariable binary logistic regression models including calprotectin, CRP, age, and sex were analyzed to assess independent associations between predictors and outcomes. Calprotectin and CRP were used dichotomized according to the optimal cut-off points in regression analyses. All analyses were 2-tailed and a *P* value <.05 was considered statistically significant. All statistical analyses were computed with IBM SPSS Statistics version 30.0.0.0.

## RESULTS

### Study Population

The original ED-Sepsis cohort included 647 patients. In the present study, 63 patients with incomplete data and 1 patient with erroneous value for temperature (8.6) were excluded from analysis. Baseline characteristics of the 583 included patients are presented in Table 1. Eighteen patients were missing information on 1 MEWS parameter, and 1 patient was missing information on 2 MEWS parameters. There was a statistically significant correlation between calprotectin and CRP (ρ = 0.56, *P* < .001). There were no statistically significant correlations between age and calprotectin or CRP. There were no statistically significant differences in median level of calprotectin or CRP between men and women.

**Table 1.** Baseline Characteristics of All Included Patients (N = 583)

Variable	All Patients	Missing Values
Age, y	69 ± 19	0
Female sex, No. (%)	284 (49)	0
Comorbidities, No. (%)		
Coronary artery disease	114 (20)	1
Heart failure	109 (19)	2
Hypertension	237 (41)	0
COPD	108 (19)	2
Renal disease	43 (7)	5
Diabetes mellitus	111 (19)	0
Obesity (BMI >30 kg/m^2^)	115 (21)	24
Cancer	162 (28)	1
Rheumatic disease	38 (7)	1
Immunodeficiency	30 (5)	7
Clinical parameters		
Respiratory rate, breaths/min	28 ± 8	4
Oxygen saturation, %, median (IQR)	93 (88–96)	3
Heart rate, beats/min	106 ± 22	1
Systolic blood pressure, mm Hg	136 ± 27	5
Temperature, °C	38.8 ± 0.9	0
MEWS (points)	5 ± 2	0
MEWS ≥5, No. (%)	339 (58)	0
Calprotectin, mg/L, median (IQR)	1.03 (0.61–2.19)	0
CRP, mg/L, median (IQR)	72 (24–163)	0

Results are shown as mean ± standard deviation unless otherwise indicated.

Abbreviations: BMI, body mass index; COPD, chronic obstructive pulmonary disease; CRP, C-reactive protein; MEWS, Modified Early Warning Score.

### Sepsis Versus Noninfectious SIRS

Of the 583 included patients, 575 received a diagnosis. Among these, 529 (92%) received an infectious diagnosis and 46 (8%) received a noninfectious diagnosis. Plasma concentrations of calprotectin (median [IQR], 1.07 [0.63–2.34] vs 0.69 [0.33–1.24]; *P* = .001) and CRP (median [IQR], 76 [30–168] vs 18 [7–38]; *P* < .001) were significantly higher in patients with sepsis compared to patients with noninfectious SIRS. There were no statistically significant differences between the groups regarding total number of MEWS points (mean ± SD, 5 ± 2 vs 5 ± 2; *P* = .211) or the proportion of patients with MEWS ≥5 (n = 310 [59%] vs n = 23 [50%]; *P* = .257). Baseline characteristics are presented in Supplementary Table 2.

In ROC analysis, MEWS did not show any discriminatory value. Calprotectin demonstrated an AUC of 0.63 (95% confidence interval [CI], .55–.71; *P* = .002) at the optimal cut-off ≥1.015 mg/L and CRP an AUC of 0.74 (95% CI, .66–.81; *P* < .001) at the optimal cut-off ≥35.5 mg/L. The addition of calprotectin to CRP did not significantly increase the AUC compared to CRP alone. In multivariable logistic regression analysis, only CRP was an independent predictor of sepsis (odds ratio, 7.26 [95% CI, 3.37–15.65]; *P* < .001).

### Source of Infection

Among the 583 included patients, information about the source of infection was available for 528. Of these 194 (37%) had LRTI, 52 (10%) had URTI, 125 (24%) had UTI, and 157 (30%) had other sources of infection. The plasma concentration of calprotectin was significantly higher in patients with LRTI than patients with URTI and UTI (Table 2). The plasma concentration of CRP was significantly higher in patients with LRTI than patients with URTI. Baseline characteristics are presented in Supplementary Table 3.

**Table 2.** Biomarker Levels According to Source of Infection

Biomarker	LRTI (n = 194)	URTI (n = 52)	UTI (n = 125)	Other (n = 157)
Calprotectin, mg/L	1.44^a^ (0.81–3.13)	0.59 (0.32–1.02)	0.87 (0.61–1.68)	1.07 (0.59–2.67)
CRP, mg/L	97^b^ (46–201)	31 (13–55)	71 (24–163)	82 (33–172)

Results are shown as median (interquartile range). Differences between groups were tested by Kruskal–Wallis 1-way analysis of variance with Bonferroni correction for multiple tests.

Abbreviations: CRP, C-reactive protein; LRTI, lower respiratory tract infection; URTI, upper respiratory tract infection; UTI, urinary tract infection.

^a^ *P* < .001 for LRTI vs URTI and LRTI vs UTI; *P* = .079 for LRTI vs other.

^b^ *P* < .001 for LRTI vs URTI; *P* = .110 for LRTI vs UTI; and *P* = 1.000 for LRTI vs other.

In ROC analysis, both calprotectin and CRP showed statistically significant discriminatory abilities across all outcomes (Table 3). Addition of calprotectin to CRP significantly increased the discriminatory ability compared to CRP alone for LRTI versus URTI (AUC, 0.76 vs 0.81; *P* = .002) and LRTI versus UTI (AUC, 0.59 vs 0.65; *P* = .007), but not for LRTI versus other sources of infection (AUC, 0.56 vs 0.60; *P* = .062). In multivariable logistic regression analysis, calprotectin was an independent predictor across all outcomes while CRP was only an independent predictor of LRTI versus URTI (Table 4).

**Table 3.** Receiver Operating Characteristic Analysis

Source of Infection	Cut-off, mg/L	AUC (95% CI)	*P* Value
Calprotectin			
LRTI vs URTI	≥0.805	0.72 (.64–.80)	<.001
LRTI vs UTI	≥1.275	0.63 (.57–.69)	<.001
LRTI vs other	≥0.805	0.57 (.51–.63)	.017
CRP			
LRTI vs URTI	≥72.5	0.76 (.69–.83)	<.001
LRTI vs UTI	≥72.5	0.59 (.52–.65)	.008
LRTI vs other	≥74.5	0.56 (.50–.62)	.046

Receiver operating characteristic analysis of LRTI and different sources of infection. Cut-off points were identified by Youden index.

Abbreviations: AUC, area under the curve; CI, confidence interval; CRP, C-reactive protein; LRTI, lower respiratory tract infection; URTI, upper respiratory tract infection; UTI, urinary tract infection.

**Table 4.** Multivariable Logistic Regression

Source of Infection	OR (95% CI)	*P* Value
LRTI vs URTI		
Calprotectin ≥0.805	3.25 (1.51–6.98)	.003
CRP ≥72.5	8.38 (3.32–21.13)	<.001
Sex	1.74 (.84–3.62)	.134
Age	1.02 (1.00–1.05)	.016
LRTI vs UTI		
Calprotectin ≥1.275	2.47 (1.48–4.12)	<.001
CRP ≥72.5	1.55 (.94–2.57)	.086
Sex	1.30 (.81–2.08)	.283
Age	1.00 (.99–1.01)	.937
LRTI vs other		
Calprotectin ≥0.805	1.74 (1.06–2.85)	.029
CRP ≥74.5	1.43 (.90–2.29)	.134
Sex	1.32 (.85–2.05)	.224
Age	1.02 (1.01–1.03)	<.001

Binary logistic regression analysis of LRTI and different sources of infection including calprotectin (dichotomized at the respective optimal cut-off points), CRP (dichotomized at the respective optimal cut-off points), sex (male), and age (years). The cut-off points for calprotectin and CRP were identified by Youden index and are expressed in mg/L.

Abbreviations: CI, confidence interval; CRP, C-reactive protein; LRTI, lower respiratory tract infection; OR, odds ratio; URTI, upper respiratory tract infection; UTI, urinary tract infection.

## DISCUSSION

In this cohort of unselected ED patients with suspected sepsis, addition of calprotectin to CRP improved the diagnostic performance for LRTI compared to CRP alone. The combination showed a good discriminatory ability for LRTI versus URTI and a modest discriminatory ability for LRTI versus UTI. Although calprotectin was inferior to CRP for sepsis detection, it shows potential as a biomarker for the source of infection.

Comparison of sepsis studies are inherently complicated as study settings, populations, and definitions differ. MEWS, originally developed to identify hospitalized medical patients at risk of deterioration [15], has AUCs of 0.72–0.85 for sepsis screening in the ED [7, 16]. In our cohort, MEWS did not show any diagnostic value, likely due to the overlap between SIRS and MEWS and differences in study populations. Calprotectin also exhibits varying diagnostic accuracies between studies. Christensen et al reported an AUC of 0.78 in patients assessed by sepsis or medical rapid response teams in the ED [17]. Diehl-Wiesenecker et al found calprotectin to have an AUC of 0.90 for differentiating patients with and without bacterial infections in the ED [18]. In contrast to these studies, we only included patients with suspected infection, which may explain the lower diagnostic accuracy in our study. On the other hand, studies in the ICU setting have reported AUCs of 0.61–0.67 [19, 20], similar to our results. Analytical qualities also complicate interstudy comparisons, as calprotectin concentration is dependent on sample type [21]. For example, the use of lithium heparin plasma as opposed to EDTA plasma could explain the higher optimal cut-off (≥1.96 mg/L vs ≥1.015 mg/L) in the study by Christensen et al [17]. Compared to calprotectin, CRP exhibits more consistent diagnostic accuracies across studies with AUCs in the 0.70s [19, 22–24], although some studies have reported AUCs <0.60 or >0.90 [17, 18, 25]. Together with CRP, procalcitonin (PCT) is the most studied and used diagnostic biomarker for sepsis [26]. PCT has been considered superior to CRP in the ICU [27], but less clearly so in the ED [28]. PCT is not routinely analyzed in our ED and could therefore not be evaluated. In this study, the comparison of calprotectin and CRP must be made with caution. The fact that CRP was available during the retrospective classification of sepsis means that the performance of CRP may be overestimated. The small proportion (8%) of patients with noninfectious SIRS also merits a cautious interpretation of the results. With these considerations in mind, the results indicate that calprotectin might not meaningfully improve sepsis detection beyond existing biomarkers. Due to the complexity of the host immune response and the heterogeneity of sepsis, elucidating the mechanisms behind biomarker accuracies is a huge challenge. Indeed, Pierrakos et al stated that biomarkers must be studied in specific clinical settings rather than as general markers of sepsis [26].

Determining the source of infection is fundamental to sepsis management as it guides antibiotic therapy [29]. Misclassification risks both over- and undertreatment, highlighting the need for reliable biomarkers. Previous research of the ability of calprotectin and CRP to discriminate the source of infection in the ED is sparse. CRP discriminates well between bacterial and viral LRTI (AUC, 0.82) [30], but poorly between UTI and other infections (AUC, 0.60) [31]. Calprotectin can discriminate between bacterial sepsis and viral infections [32] and identify infections in patients with acute dyspnea [33]. In a cohort of ICU patients with severe sepsis, those with pneumonia as the source of infection had the highest serum calprotectin concentration [11]. Our results support the previous findings that CRP is a useful discriminator within respiratory infections but a poor one for UTI. Calprotectin showed a similar discriminatory ability as CRP within respiratory infections and a modest ability for LRTI versus UTI. Notably, addition of calprotectin to CRP significantly improved the discriminatory ability for LRTI versus URTI and LRTI versus UTI. Furthermore, calprotectin was an independent predictor for both outcomes while CRP was only an independent predictor of LRTI versus URTI. This suggests that calprotectin may add clinically relevant information when the differential diagnosis includes both respiratory and urinary tract infections—the 2 most common sources of infection in sepsis [29]. Neutrophil activation, including release of calprotectin, is one of the first steps in the immune response against infection [10]. Calprotectin is involved in several neutrophil actions, such as migration, respiratory burst, degranulation, and formation of neutrophil extracellular traps [34]. While these antimicrobial actions are highly effective, excessive or abnormal neutrophil activation can cause tissue damage leading to organ dysfunction [35], including acute lung injury and acute respiratory distress syndrome (ARDS) in sepsis [36]. The excessive release of calprotectin can then form a positive feedback loop contributing to hyperinflammation [34]. Hence, calprotectin could be a marker of a dysregulated neutrophil response. In support of this, clinical studies have shown an association between calprotectin and severity of coronavirus disease 2019 (COVID-19) [37] and development of ARDS in septic ICU patients [38]. In an experimental model of COVID-19, blockade of calprotectin improved survival and reduced pulmonary neutrophil invasion and damage [39]. Similarly, blockade of S100A9 reduced pulmonary neutrophil invasion and damage in an experimental model of abdominal sepsis [40]. Collectively, these results highlight the need for further clinical and mechanistic research of calprotectin in pulmonary infections and sepsis.

### Clinical Implications

Early antibiotic therapy is fundamental to improving sepsis outcome. Empirical use of broad-spectrum agents is appealing from a pathogen coverage perspective but contributes to antibiotic resistance [3]. Our results suggest that calprotectin can improve discrimination between the most common sources of infection in ED patients with sepsis and should be validated in additional studies, including comparison with PCT. To further explore the clinical utility of calprotectin, future studies should investigate if calprotectin can aid ED physicians in directing antibiotic therapy without loss of appropriate coverage. In these studies, the choice of sample type must be considered as it affects the clinical relevance and comparability of results.

### Limitations and Strengths

The study has several limitations. First, the single-center design limits generalizability to other settings. Second, sepsis was defined according to Sepsis-2 [5], which has since been replaced by Sepsis-3 [4]. This limits generalizability in the setting of Sepsis-3; however, SIRS is a more sensitive screening tool for sepsis than qSOFA [6]. Third, CRP was measured at ED arrival according to local practice and the results were available when reviewing the medical records for final diagnosis. In an ideal scenario, both biomarkers would be blinded; however, a novel biomarker should be compared to one already implemented into clinical practice. Fourth, CRP and PCT are the most studied and used diagnostic biomarkers for sepsis. In this study, only CRP was available for comparison. Fifth, 92% of the patients had sepsis. Inclusion of patients without suspicion of infection would have made the groups more even but would also have made the results less clinically relevant. Sixth, the cut-off points identified by Youden index are optimal from a statistical point of view but not necessarily from a clinical one. Unfortunately, there are no established clinical cut-off points for the outcomes of this study.

The study also has several strengths. It includes a large number of prospectively included patients that are well characterized. The few exclusion criteria, and inclusion based on clinical suspicion of sepsis, makes the cohort representative of a “real-world” ED population. Finally, we believe that retrospective diagnosis by chart review provides a more accurate classification compared to computerized searches of coded discharge diagnoses.

## CONCLUSIONS

Calprotectin may improve differentiation of lower respiratory tract infections from upper respiratory tract infections and urinary tract infections in patients presenting to the ED with sepsis. Further research is needed to clarify if calprotectin adds diagnostic value to standard clinical assessment.

## Supplementary Material

ofag331_Supplementary_Data

## Supplementary Data

Supplementary materials are available at *Open Forum Infectious Diseases* online. Consisting of data provided by the authors to benefit the reader, the posted materials are not copyedited and are the sole responsibility of the authors, so questions or comments should be addressed to the corresponding author.
