# Supplementary material for: Calprotectin as a Diagnostic Marker for Lower Respiratory Tract Infection and Sepsis in the Emergency Department
Source: Open Forum Infect Dis. 2026 Jun 3;13(6):ofag331. doi: 10.1093/ofid/ofag331 (PMC13251339; doi:10.1093/ofid/ofag331)
Supplement: ofag331_Supplementary_Data [file ofag331_supplementary_data.zip › Supplementary Table 1.docx]

Supplementary Table 1. Distribution of the different sources of infection (N = 528).

| LRTI | 194 (37) |
| --- | --- |
| URTI | 52 (10) |
| UTI | 125 (24) |
| Skin and soft tissue | 56 (11) |
| Abdominal | 21 (4) |
| Bone and joint | 7 (1) |
| Implantable device | 5 (1) |
| Vascular catheter | 5 (1) |
| Endocarditis | 2 (0) |
| Other | 45 (9) |
| Unknown | 16 (3) |

Results are shown as N (%).

*LRTI* Lower respiratory tract infection, *URTI* Upper respiratory tract infection, *UTI* Urinary tract infection
