# Supplementary material for: Calprotectin as a Diagnostic Marker for Lower Respiratory Tract Infection and Sepsis in the Emergency Department
Source: Open Forum Infect Dis. 2026 Jun 3;13(6):ofag331. doi: 10.1093/ofid/ofag331 (PMC13251339; doi:10.1093/ofid/ofag331)
Supplement: ofag331_Supplementary_Data [file ofag331_supplementary_data.zip › Supplementary Table 2.docx]

Supplementary Table 2. Baseline characteristics according to infection status.

|  | **No infection**  **N=46** | **Infection**  **N=529** | **Missing values** |
| --- | --- | --- | --- |
| **Age (years)** | 66 (±18) | 69 (±19) | 0 |
| **Female** | 27 (59) | 252 (48) | 0 |
| **Comorbidities** |  |  |  |
| Coronary artery disease | 7 (16) | 105 (20) | 1 |
| Heart failure | 11 (24) | 96 (18) | 2 |
| Hypertension | 19 (41) | 217 (41) | 0 |
| COPD | 10 (22) | 97 (18) | 2 |
| Renal disease | 1 (2) | 41 (8) | 5 |
| Diabetes mellitus | 13 (28) | 97 (18) | 0 |
| Obesity (BMI > 30 kg/m^2^) | 16 (36) | 97 (19) | 24 |
| Cancer | 6 (13) | 155 (29) | 1 |
| Rheumatic disease | 5 (11) | 32 (6) | 1 |
| Immunodeficiency | 1 (2) | 29 (6) | 7 |
| **Clinical parameters** |  |  |  |
| Respiratory rate (breaths/min) | 27 (±8) | 28 (±8) | 4 |
| Oxygen saturation (%) | 92 (87 – 96) | 93 (88 – 96) | 3 |
| Heart rate (beats/min) | 110 (±18) | 106 (±22) | 1 |
| Systolic blood pressure (mmHg) | 143 (±28) | 136 (±27) | 5 |
| Temperature (Celsius) | 38.5 (±0.9) | 38.8 (±0.9) | 0 |

Results are shown as Mean (±SD), Median (IQR) or Number (%).

*COPD* Chronic obstructive pulmonary disease, *BMI* Body mass index.
