# Supplementary material for: Calprotectin as a Diagnostic Marker for Lower Respiratory Tract Infection and Sepsis in the Emergency Department
Source: Open Forum Infect Dis. 2026 Jun 3;13(6):ofag331. doi: 10.1093/ofid/ofag331 (PMC13251339; doi:10.1093/ofid/ofag331)
Supplement: ofag331_Supplementary_Data [file ofag331_supplementary_data.zip › Supplementary Table 3.docx]

Supplementary Table 3. Baseline characteristics according to source of infection.

|  | **LRTI**  **N = 194** | **URTI**  **N = 52** | **UTI**  **N = 125** | **Other**  **N = 157** | **Missing values** |
| --- | --- | --- | --- | --- | --- |
| **Age (years)** | 71 (±18) | 65 (±18) | 72 (±17) | 64 (±20) | 0 |
| **Female** | 85 (44) | 28 (54) | 63 (50) | 75 (48) | 0 |
| **Comorbidities** |  |  |  |  |  |
| Coronary artery disease | 42 (22) | 7 (14) | 29 (23) | 26 (17) | 0 |
| Heart failure | 43 (22) | 9 (17) | 19 (15) | 25 (16) | 2 |
| Hypertension | 81 (42) | 20 (39) | 58 (46) | 57 (36) | 0 |
| COPD | 53 (28) | 16 (31) | 13 (10) | 15 (10) | 2 |
| Renal disease | 13 (7) | 5 (10) | 11 (9) | 12 (8) | 5 |
| Diabetes mellitus | 28 (14) | 10 (19) | 27 (22) | 32 (20) | 0 |
| Obesity (BMI > 30 kg/m^2^) | 27 (15) | 11 (22) | 26 (22) | 33 (22) | 22 |
| Cancer | 62 (32) | 13 (25) | 36 (29) | 44 (28) | 1 |
| Rheumatic disease | 15 (8) | 2 (4) | 7 (6) | 8 (5) | 1 |
| Immunodeficiency | 13 (7) | 0 (0) | 6 (5) | 10 (7) | 7 |
| **Clinical parameters** |  |  |  |  |  |
| Respiratory rate (breaths/min) | 30 (±8) | 26 (±7) | 27 (±7) | 26 (±8) | 4 |
| Oxygen saturation (%) | 90 (83 – 94) | 93 (88 – 97) | 94 (90 – 97) | 95 (92 – 98) | 3 |
| Heart rate (beats/min) | 107 (±22) | 99 (±19) | 107 (±23) | 106 (±22) | 1 |
| Systolic blood pressure (mmHg) | 136 (±29) | 136 (±22) | 136 (±27) | 135 (±25) | 5 |
| Temperature (Celsius) | 38.7 (±0.9) | 38.6 (±0.7) | 39.0 (±0.9) | 38.8 (±0.9) | 0 |

Results are shown as Mean (±SD), Median (IQR) or Number (%).

*COPD* Chronic obstructive pulmonary disease, *BMI* Body mass index.
